# 2-Phenyl-4*H*-3,1-benzoxazin-4-one

**DOI:** 10.1107/S1600536808042050

**Published:** 2008-12-17

**Authors:** R. Thilagavathy, Helen P. Kavitha, R. Arulmozhi, Jasmine P. Vennila, V. Manivannan

**Affiliations:** a Department of Chemistry, SRM University, Ramapuram Campus, Chennai 600 089, India; b Department of Chemistry, SRM University, Kattankulathur Campus, Kanchipuram, India; c Department of Physics, Panimalar Institute of Technology, Chennai, India; d Department of Physics, Presidency College, Chennai 600 005, India

## Abstract

The title mol­ecule, C_14_H_9_NO_2_, is nearly planar with a dihedral angle of 3.72 (4)° beteewn the plane of the phenyl ring and the 3,1-benzoxazin-4-one fragment. The mol­ecules are arranged into stacks parallel to the *b* axis *via* π–π stacking inter­actions [centroid-centroid distance = 4.2789 (11) Å] and the crystal packing is additionally stabilized by weak inter­molecular C—H⋯O inter­actions.

## Related literature

For the biological activity of oxazin-4-ones, see: Pietsch & Gütschow (2005 ▶); Tarzia *et al.* (2007 ▶). For similar structures, see: Crane & Rogerson (2004 ▶); Khan *et al.* (2007 ▶).
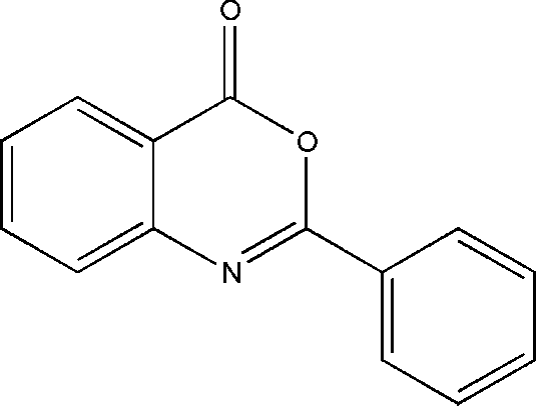


## Experimental

### 

#### Crystal data

C_14_H_9_NO_2_
*M* *_r_* = 223.22
Monoclinic, 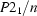

*a* = 13.3055 (16) Å
*b* = 3.8930 (4) Å
*c* = 20.445 (2) Å
β = 94.946 (3)°
*V* = 1055.1 (2) Å^3^
*Z* = 4
Mo *K*α radiation
μ = 0.10 mm^−1^
*T* = 295 (2) K
0.20 × 0.16 × 0.16 mm

#### Data collection

Bruker Kappa APEXII diffractometer
Absorption correction: multi-scan (*SADABS*; Sheldrick, 1996 ▶) *T*_min_ = 0.981, *T*_max_ = 0.985
13688 measured reflections
3034 independent reflections
1800 reflections with *I* > 2σ(*I*)
*R*_int_ = 0.036

#### Refinement

*R*[*F* ^2^ > 2σ(*F* ^2^)] = 0.053
*wR*(*F* ^2^) = 0.187
*S* = 1.08
3034 reflections
154 parameters
H-atom parameters constrained
Δρ_max_ = 0.24 e Å^−3^
Δρ_min_ = −0.28 e Å^−3^

### 

Data collection: *APEX2* (Bruker, 2004 ▶); cell refinement: *SAINT* (Bruker, 2004 ▶); data reduction: *SAINT*; program(s) used to solve structure: *SHELXS97* (Sheldrick, 2008 ▶); program(s) used to refine structure: *SHELXL97* (Sheldrick, 2008 ▶); molecular graphics: *PLATON* (Spek, 2003 ▶); software used to prepare material for publication: *SHELXL97*.

## Supplementary Material

Crystal structure: contains datablocks global, I. DOI: 10.1107/S1600536808042050/gk2180sup1.cif

Structure factors: contains datablocks I. DOI: 10.1107/S1600536808042050/gk2180Isup2.hkl

Additional supplementary materials:  crystallographic information; 3D view; checkCIF report

## Figures and Tables

**Table 1:** Hydrogen-bond geometry (Å, °)

*D*—H⋯*A*	*D*—H	H⋯*A*	*D*⋯*A*	*D*—H⋯*A*
C10—H10⋯O2^i^	0.93	2.51	3.294 (2)	142

Symmetry code: (i) 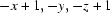.

**Fig. 1.**
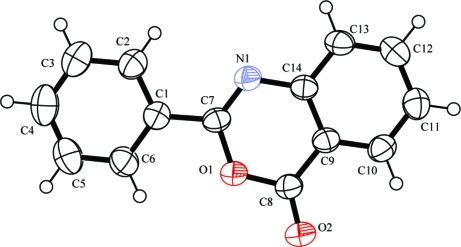
The molecular structure of the title compound, with atom labels and 50% probability displacement ellipsoids for non-H atoms.

**Fig. 2.**
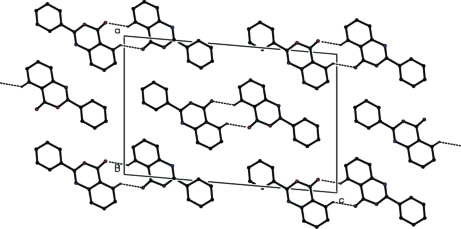
The crystal packing viewed down the b axis. C-H···O hydrogen bonds are shown as dashed lines.

## supplementary crystallographic information

### Comment

Oxazin-4-one derivatives are used as inhibitors of the alpha/beta hydrolases, cholesterol esterase and acetylcholinesterase (Pietsch & Gütschow, 2005) and are potent inhibitors of the endocannabinoid-deactivating enzyme, monoacylglycerol lipase (Tarzia *et al.*, 2007).

The geometric parameters of the title molecule (Fig. 1) agree well with the earlier reported structures (Crane & Rogerson, 2004; Khan *et al.*, 2007). The plane of the phenyl ring forms a dihedral angle of 3.72 (4)° with the benzo[*d*][1,3]oxazin-4-one moiety. The molecular structure is stabilized by weak intramolecular C–H···O interaction and the crystal packing is stabilized by weak intermolecular C—H···O and π-π stacking interactions.

### Experimental

To a stirred solution of anthranilic acid (0.01 mol) in pyridine (60 ml), benzoyl chloride (0.01 mol) was added dropwise maintaining the temperature near 8° C for one hour. The reaction mixture was stirred for another 2 h at room temperature. While stirring, a solid product separated out. The whole reaction mixture was neutralized with NaHCO_3_ solution. A pale yellow solid deposited was filtered, washed with water and recrystallized from ethanol to get diffraction quality crystals; yYield 78%.

### Refinement

H atoms were positioned geometrically and refined using riding model with C—H = 0.93Å and *U*_iso_(H) = 1.2Ueq(C).

### Crystal data

**Table:**

C_14_H_9_NO_2_	*F*(000) = 464
*M**_r_* = 223.22	*D*_x_ = 1.405 Mg m^−^^3^
Monoclinic, *P*2_1_/*n*	Mo *K*α radiation, λ = 0.71073 Å
Hall symbol: -P 2yn	Cell parameters from 2312 reflections
*a* = 13.3055 (16) Å	θ = 1.7–29.5°
*b* = 3.8930 (4) Å	µ = 0.10 mm^−^^1^
*c* = 20.445 (2) Å	*T* = 295 K
β = 94.946 (3)°	Block, pale yellow
*V* = 1055.1 (2) Å^3^	0.20 × 0.16 × 0.16 mm
*Z* = 4	

### Data collection

**Table:**

Bruker Kappa APEXII diffractometer	3034 independent reflections
Radiation source: fine-focus sealed tube	1800 reflections with *I* > 2σ(*I*)
graphite	*R*_int_ = 0.036
Detector resolution: 0 pixels mm^-1^	θ_max_ = 29.8°, θ_min_ = 1.8°
ω and φ scans	*h* = −18→17
Absorption correction: multi-scan (*SADABS*; Sheldrick, 1996)	*k* = −5→5
*T*_min_ = 0.981, *T*_max_ = 0.985	*l* = −28→28
13688 measured reflections	

### Refinement

**Table:**

Refinement on *F*^2^	Primary atom site location: structure-invariant direct methods
Least-squares matrix: full	Secondary atom site location: difference Fourier map
*R*[*F*^2^ > 2σ(*F*^2^)] = 0.053	Hydrogen site location: inferred from neighbouring sites
*wR*(*F*^2^) = 0.187	H-atom parameters constrained
*S* = 1.08	*w* = 1/[σ^2^(*F*_o_^2^) + (0.0948*P*)^2^] where *P* = (*F*_o_^2^ + 2*F*_c_^2^)/3
3034 reflections	(Δ/σ)_max_ < 0.001
154 parameters	Δρ_max_ = 0.24 e Å^−^^3^
0 restraints	Δρ_min_ = −0.28 e Å^−^^3^

### Fractional atomic coordinates and isotropic or equivalent isotropic displacement parameters (Å^2^)

**Table:**

	*x*	*y*	*z*	*U*_iso_*/*U*_eq_	
C1	0.54301 (13)	0.4961 (4)	0.20378 (8)	0.0439 (4)	
C2	0.48811 (15)	0.6405 (4)	0.15009 (9)	0.0533 (5)	
H2	0.4227	0.7175	0.1539	0.064*	
C3	0.52987 (17)	0.6703 (5)	0.09133 (10)	0.0663 (6)	
H3	0.4924	0.7639	0.0551	0.080*	
C4	0.62706 (19)	0.5616 (6)	0.08600 (11)	0.0718 (6)	
H4	0.6554	0.5844	0.0462	0.086*	
C5	0.68254 (16)	0.4204 (5)	0.13861 (12)	0.0680 (6)	
H5	0.7482	0.3472	0.1345	0.082*	
C6	0.64107 (13)	0.3866 (5)	0.19766 (10)	0.0549 (5)	
H6	0.6787	0.2904	0.2335	0.066*	
C7	0.49642 (12)	0.4637 (4)	0.26569 (8)	0.0412 (4)	
C8	0.52751 (12)	0.2379 (4)	0.37481 (8)	0.0466 (4)	
C9	0.42925 (12)	0.3731 (4)	0.38655 (8)	0.0412 (4)	
C10	0.39216 (13)	0.3387 (4)	0.44755 (9)	0.0489 (4)	
H10	0.4307	0.2312	0.4817	0.059*	
C11	0.29830 (14)	0.4643 (5)	0.45710 (9)	0.0534 (5)	
H11	0.2733	0.4445	0.4980	0.064*	
C12	0.24090 (14)	0.6201 (5)	0.40597 (10)	0.0535 (5)	
H12	0.1771	0.7027	0.4128	0.064*	
C13	0.27615 (12)	0.6554 (4)	0.34535 (9)	0.0479 (4)	
H13	0.2364	0.7599	0.3113	0.057*	
C14	0.37202 (12)	0.5334 (4)	0.33502 (8)	0.0404 (4)	
N1	0.40839 (10)	0.5770 (3)	0.27363 (7)	0.0441 (4)	
O1	0.55759 (8)	0.2978 (3)	0.31294 (6)	0.0495 (3)	
O2	0.58392 (10)	0.0790 (4)	0.41188 (6)	0.0698 (4)	

### Atomic displacement parameters (Å^2^)

**Table:**

	*U*^11^	*U*^22^	*U*^33^	*U*^12^	*U*^13^	*U*^23^
C1	0.0438 (9)	0.0438 (8)	0.0439 (10)	−0.0068 (7)	0.0027 (7)	−0.0010 (7)
C2	0.0560 (11)	0.0553 (10)	0.0484 (11)	−0.0045 (8)	0.0033 (9)	0.0046 (8)
C3	0.0816 (16)	0.0675 (12)	0.0503 (12)	−0.0070 (10)	0.0083 (11)	0.0089 (9)
C4	0.0859 (17)	0.0717 (13)	0.0618 (14)	−0.0187 (11)	0.0290 (12)	−0.0008 (10)
C5	0.0567 (12)	0.0758 (13)	0.0743 (15)	−0.0074 (10)	0.0225 (11)	−0.0052 (11)
C6	0.0443 (10)	0.0630 (11)	0.0578 (12)	−0.0049 (8)	0.0065 (9)	0.0011 (8)
C7	0.0384 (9)	0.0414 (8)	0.0422 (10)	−0.0032 (6)	−0.0053 (7)	0.0008 (6)
C8	0.0404 (9)	0.0546 (9)	0.0440 (10)	0.0025 (7)	−0.0012 (8)	0.0069 (7)
C9	0.0391 (9)	0.0418 (8)	0.0415 (9)	−0.0023 (6)	−0.0027 (7)	−0.0003 (6)
C10	0.0496 (10)	0.0531 (9)	0.0431 (10)	−0.0017 (7)	−0.0018 (8)	0.0040 (7)
C11	0.0542 (11)	0.0592 (10)	0.0476 (11)	−0.0037 (8)	0.0100 (9)	−0.0048 (8)
C12	0.0424 (10)	0.0569 (10)	0.0618 (13)	0.0010 (7)	0.0071 (9)	−0.0099 (8)
C13	0.0393 (9)	0.0531 (9)	0.0499 (11)	0.0030 (7)	−0.0047 (8)	−0.0020 (7)
C14	0.0370 (8)	0.0408 (8)	0.0423 (9)	−0.0020 (6)	−0.0019 (7)	−0.0022 (6)
N1	0.0399 (8)	0.0495 (7)	0.0421 (8)	0.0008 (6)	−0.0018 (6)	0.0033 (6)
O1	0.0387 (7)	0.0644 (7)	0.0447 (7)	0.0069 (5)	0.0003 (5)	0.0075 (5)
O2	0.0532 (8)	0.0981 (10)	0.0576 (9)	0.0236 (7)	0.0018 (7)	0.0257 (7)

### Geometric parameters (Å, °)

**Table:**

C1—C2	1.384 (2)	C8—O2	1.1926 (19)
C1—C6	1.388 (2)	C8—O1	1.3791 (19)
C1—C7	1.462 (2)	C8—C9	1.448 (2)
C2—C3	1.371 (2)	C9—C10	1.387 (2)
C2—H2	0.9300	C9—C14	1.393 (2)
C3—C4	1.374 (3)	C10—C11	1.371 (2)
C3—H3	0.9300	C10—H10	0.9300
C4—C5	1.367 (3)	C11—C12	1.381 (3)
C4—H4	0.9300	C11—H11	0.9300
C5—C6	1.376 (3)	C12—C13	1.369 (2)
C5—H5	0.9300	C12—H12	0.9300
C6—H6	0.9300	C13—C14	1.394 (2)
C7—N1	1.275 (2)	C13—H13	0.9300
C7—O1	1.3702 (18)	C14—N1	1.394 (2)
			
C2—C1—C6	119.27 (17)	O2—C8—C9	127.66 (16)
C2—C1—C7	119.16 (15)	O1—C8—C9	115.34 (14)
C6—C1—C7	121.56 (16)	C10—C9—C14	120.63 (15)
C3—C2—C1	120.20 (18)	C10—C9—C8	120.68 (15)
C3—C2—H2	119.9	C14—C9—C8	118.68 (15)
C1—C2—H2	119.9	C11—C10—C9	119.55 (16)
C2—C3—C4	119.9 (2)	C11—C10—H10	120.2
C2—C3—H3	120.0	C9—C10—H10	120.2
C4—C3—H3	120.0	C10—C11—C12	120.00 (17)
C5—C4—C3	120.64 (19)	C10—C11—H11	120.0
C5—C4—H4	119.7	C12—C11—H11	120.0
C3—C4—H4	119.7	C13—C12—C11	121.27 (16)
C4—C5—C6	119.9 (2)	C13—C12—H12	119.4
C4—C5—H5	120.0	C11—C12—H12	119.4
C6—C5—H5	120.0	C12—C13—C14	119.49 (16)
C5—C6—C1	120.05 (19)	C12—C13—H13	120.3
C5—C6—H6	120.0	C14—C13—H13	120.3
C1—C6—H6	120.0	C9—C14—N1	121.73 (14)
N1—C7—O1	124.73 (15)	C9—C14—C13	119.05 (15)
N1—C7—C1	122.90 (15)	N1—C14—C13	119.22 (15)
O1—C7—C1	112.37 (14)	C7—N1—C14	117.80 (14)
O2—C8—O1	117.00 (15)	C7—O1—C8	121.64 (12)
			
C6—C1—C2—C3	0.9 (2)	C9—C10—C11—C12	0.8 (3)
C7—C1—C2—C3	−179.33 (15)	C10—C11—C12—C13	−0.6 (3)
C1—C2—C3—C4	−1.0 (3)	C11—C12—C13—C14	−0.3 (3)
C2—C3—C4—C5	0.7 (3)	C10—C9—C14—N1	178.74 (14)
C3—C4—C5—C6	−0.1 (3)	C8—C9—C14—N1	−2.3 (2)
C4—C5—C6—C1	0.0 (3)	C10—C9—C14—C13	−0.8 (2)
C2—C1—C6—C5	−0.4 (3)	C8—C9—C14—C13	178.16 (14)
C7—C1—C6—C5	179.88 (15)	C12—C13—C14—C9	1.0 (2)
C2—C1—C7—N1	−3.3 (2)	C12—C13—C14—N1	−178.56 (14)
C6—C1—C7—N1	176.50 (15)	O1—C7—N1—C14	0.9 (2)
C2—C1—C7—O1	176.31 (13)	C1—C7—N1—C14	−179.59 (12)
C6—C1—C7—O1	−3.9 (2)	C9—C14—N1—C7	0.2 (2)
O2—C8—C9—C10	3.1 (3)	C13—C14—N1—C7	179.81 (15)
O1—C8—C9—C10	−177.93 (14)	N1—C7—O1—C8	0.1 (2)
O2—C8—C9—C14	−175.84 (17)	C1—C7—O1—C8	−179.43 (13)
O1—C8—C9—C14	3.1 (2)	O2—C8—O1—C7	176.92 (16)
C14—C9—C10—C11	−0.1 (2)	C9—C8—O1—C7	−2.1 (2)
C8—C9—C10—C11	−179.02 (15)		

### Hydrogen-bond geometry (Å, °)

**Table:**

*D*—H···*A*	*D*—H	H···*A*	*D*···*A*	*D*—H···*A*
C6—H6···O1	0.93	2.39	2.713 (2)	101
C10—H10···O2^i^	0.93	2.51	3.294 (2)	142

Symmetry codes: (i) −*x*+1, −*y*, −*z*+1.
